# Rapid Assessment of the Toxicity of Fungal Compounds Using Luminescent *Vibrio qinghaiensis* sp. Q67

**DOI:** 10.3390/toxins9100335

**Published:** 2017-10-21

**Authors:** Qijie Jian, Liang Gong, Taotao Li, Yong Wang, Yu Wu, Feng Chen, Hongxia Qu, Xuewu Duan, Yueming Jiang

**Affiliations:** 1 Key Laboratory of Plant Resource Conservation and Sustainable Utilization, Guangdong Provincial Key Laboratory of Applied Botany, South China Botanical Garden, Chinese Academy of Sciences, Guangzhou 510650, China; jianqijie@scbg.ac.cn (Q.J.); lianggong@scbg.ac.cn (L.G.); wubangcai88@163.com (T.L.); 18255137100@163.com (Y.W.); q-hxia@scbg.ac.cn (H.Q.); xwduan@scib.ac.cn (X.D.); 2 School of Life Sciences, University of Chinese Academy of Sciences, Beijing 100039, China; 3 Zhongshan Entry-Exit Inspection and Quarantine Bureau, Zhongshan 528403, China; wrone@163.com; 4 Department of Food, Nutrition and Packaging Sciences, Clemson University, Clemson, South Carolina, SC 29634, USA; fchen@clemson.edu

**Keywords:** toxicity, mycotoxin, bioluminescence, *Vibrio qinghaiensis* sp. Q67

## Abstract

Most tropical fruits after harvest are very perishable because of fungal infection. Since some pathogenic fungi can produce hazardous compounds such as mycotoxins, novel rapid and effective methods to assess those hazardous compounds are urgently needed. Herein we report that *Vibrio qinghaiensis* sp. Q67, a luminescent bacterium, can be used to rapidly assess the toxicities of mycotoxins and cultures from mycotoxin-producing pathogens. A good correlation (*R*^2^ > 0.98) between concentrations of the mycotoxins (fumonisin B1, deoxynivalenol, zearalenone, ochratoxin A, patulin, and citrinin) and the luminous intensity of *V. qinghaiensis* sp. Q67 was obtained. Furthermore, significant correlations (*R*^2^ > 0.96) between the amount of mycotoxin and the luminous intensity from the cultures of 10 major mycotoxin-producing pathogens were also observed. In addition, *Fusarium proliferatum* (half-maximal inhibitory concentration (IC_50_) = 17.49%) exhibited greater luminescence suppression than *Fusarium semitectum* (IC_50_ = 92.56%) or *Fusarium oxysporum* (IC_50_ = 28.61%), which was in agreement with the existing higher levels of fumonisin B1, fumonisin B2, and deoxynivalenol, which were measured by high-performance liquid chromatography-tandem mass spectrometry. These results suggest that *V. qinghaiensis* sp. Q67 is a promising alternative for the rapid evaluation of the toxicity of fungal mycotoxins.

## 1. Introduction

Mycotoxins produced by fungal pathogens can extensively contaminate food and feed, causing significant risks to human and animal health. However, it is difficult to practically and effectively avoid mycotoxin contamination in natural or processed foods [1,2,3]. Particularly, ripened fruits after harvest in tropical areas are susceptible to invasion by pathogenic fungi because of the rich nutrients of the fruits and muggy climate that are suitable for fungal growth. Previous investigations have reported that many fungal pathogens isolated from tropical fruits can produce many mycotoxins [4,5]. The species in the genus *Fusarium*, including *Fusarium proliferatum*, *Fusarium semitectum*, *Fusarium oxysporum*, and *Fusarium verticillioides*, were reported to produce various mycotoxins, such as fumonisins (FBs), tricothecenes, and zearalenones (ZENs) [2]. The genus *Penicillium*, including *Penicillium citrinum* and *Penicillium expansum*, can synthesize aflatoxins, ochratoxin A (OTA), patulin, and citrinin. Although the amounts of mycotoxins are usually very low in natural foods, for example, OTA was detected in a range from 0.41 to 2.71 µg kg^−1^ in fresh fruit, and the frequent occurrence and severe toxicities of mycotoxins have been a great threat to human health [6,7]. Most of the aforementioned mycotoxins have been reported to be carcinogenic, mutagenic, teratogenic, and immunosuppressive at a concentration of ≤0.1 ppm (0.1 µg kg^−1^) [8], while the biological toxicities of those secondary compounds in some cases are unclear. Nevertheless, development of a rapid assay to evaluate the potential toxicity of all fungal products is urgently needed, especially when several mycotoxins may be simultaneously present in fresh food products. 

Currently, the major methods to analyze mycotoxins include the thin-layer chromatography (TLC), high-performance liquid chromatography (HPLC), gas chromatography–mass spectrometry (GC-MS), and enzyme-linked immunosorbent assays (ELISA). However, these methods require expensive equipment and/or reagents. Moreover, they are time-consuming, and often lack of toxicological assessments [9,10,11]. In contrast, bioassays can directly assess the toxicity of molecules. The toxicity assays of mycotoxins on different animals have been reported [12]. For example, Kouadio et al. (2013) found that the no-observed-adverse-effect level (NOAEL) of deoxynivalenol (DON) in mice was lower than 45 µg kg^−1^ day^−1^, and Nagashima and Nakagawa (2014) reported that DON hindered cell proliferation in the human promyelocytic leukemia cell line HL60 with an IC_50_ of 0.36 µg mL^−1^, although it was shown that the sensitivity of toxicity ranged from 0.1 µg mL^−1^ to 100 µg mL^−1^ in cell-based systems [12,13,14]. However, the bioassays mentioned above are often costly and complicated. In contrast, bioluminescence and chemiluminescence-based bioassays that have been used for daily detection of toxins are relatively cheaper and can be implemented more easily. Moreover, these types of assays have exhibited good sensitivities and accuracies in quantification and biological tests. 

Luminescent bacteria can serve as good indicators for toxic substrates and have been widely used in detecting pesticide residues, food additives and environmental pollution [15,16]. For example, the luminescent bacterium *Vibrio fischeri* was used to assess toxicity of three insecticidal by-products (i.e., 2-isopropyl-6-methyl-4-pyrimidinol, 3,5,6-trichloro-2-pyridinol and 6-chloronicotinic acid) [17]. Luminescent *Vibrio* species, such as *V. fischeri* and *V. harveyi*, have been also used for toxicity tests because of their natural bioluminescence properties, which are encoded by *lux* genes [18]. The luminescence reaction catalyzed by the flavine mononucleotide (FMN) oxidoreductase-luciferase is determined to be FMNH_2_ + O_2_ + R-CHO → FMN + R-COOH + H_2_O + light, which is accompanied by light emission within a blue-green wavelength of the spectrum (450–490 nm) and can be detected precisely by an ultra-weak luminescence analyzer [19]. Thus, when a luminescent bacterium is exposed to inhibitors, such as antibiotics or toxic chemicals, the inhibition of bacterial physiological activity and luciferase activity will reduce its luminescence, which can be detected via a bioluminescence analyzer. The half-maximal effective concentration (EC_50_) or a half-maximal inhibitory concentration (IC_50_), a concentration of an inhibitor that causes a 50% reduction of luminescence [20], can be calculated.

Among these reported luminescent bacteria, *Vibrio qinghaiensis* sp. Q67 that belongs to a freshwater luminescent bacterium [21] is of the greatest interest. Compared with other oceanic luminescent bacteria, its consistent performance in assessing toxicities within a wide range of ionic liquid is a very desirable feature [21,22]. Furthermore, this bacterium has been proven to be highly reliable in a wide range from pH 3.0 to 10.0, so it has been successfully used to quantify fusaric acid. *V. qinghaiensis* sp. Q67 also gives rise to strong luminescence and carries out sensitive reaction when used in detecting toxicity of chemical pollutants such as phthalate esters, phenol and aniline [23,24]. Thus, this luminescent bacterium seems to be an ideal monitor for assessing the potential toxicity under complex conditions.

The objective of this study is to rapidly assess the potential toxicity of fungal compounds using luminescent *Vibrio qinghaiensis* sp. Q67 prior to identification of these fungal compounds. In the present study, we report that *V. qinghaiensis* sp. Q67 can be used to calculate the IC_50_ values of standard mycotoxins, including FB_1_, DON, ZEN, OTA, patulin, and citrinin. Furthermore, we determine the IC_50_ values of the cultures of 10 major mycotoxin-producing pathogens isolated from rotten tropical fruits using this bacterial strain. Finally, the amount of mycotoxin production by these fungi was confirmed by HPLC-tandem mass spectrometry (MS/MS). In general, this study was conducted to develop a reliable and rapid biological assay, based on the *V. qinghaiensis* sp. Q67 as a biological tool, for determination of the toxicity of fungal compounds.

## 2. Results and Discussion

**Figure 1:**
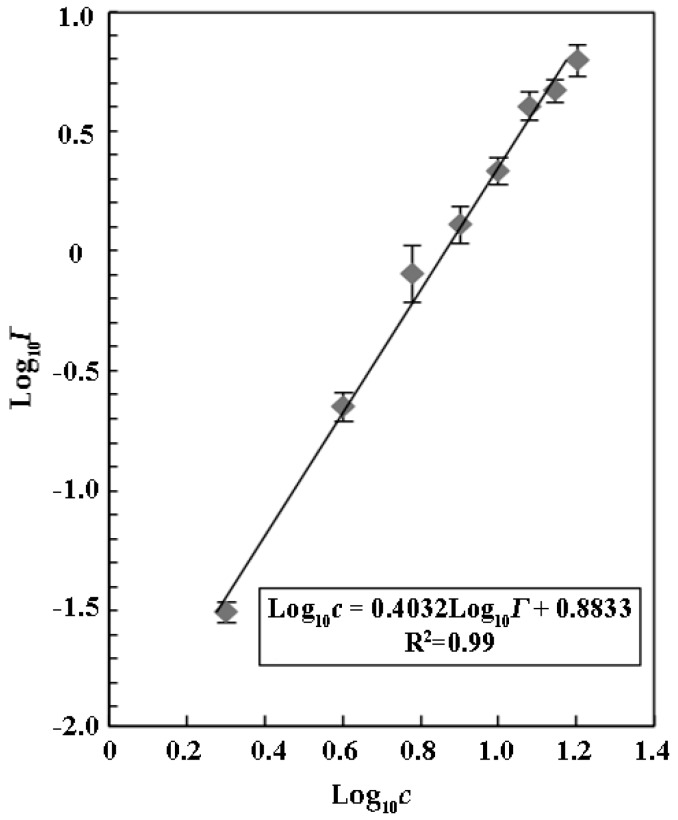
Luminescence inhibition of ZnSO_4_ solution against the *V. qinghaiensis* sp. Q67. The luminescence inhibition was enhanced with increasing concentration of the ZnSO_4_ solution.

Menz et al. (2013) reported that ZnSO_4_ could serve as a reference substrate to evaluate the sensitivity and variability of the luminescent bacterium *V. fischeri* due to its inhibitory effects on relative luminescence with the IC_50_ of 26.0 ± 3.3 mg mL^−1^ [25]. We found that ZnSO_4_ also inhibited luminescence of *V. qinghaiensis* Q67 in a concentration-dependent manner with an IC_50_ of 7.64 ± 0.42 mg mL^−1^ (Figure 1). Our results suggested that *V. qinghaiensis* Q67 was a more suitable organism than *V. fischeri* for the assays due its higher sensitivity to a luminescence inhibitor. 

*V. qinghaiensis* sp. Q67 has been used for detecting and evaluating hazardous substances, such as heavy metals and ionic liquids [26,27], but the evaluations of *V. qinghaiensis* sp. Q67 on mycotoxins and the cultures of mycotoxin-producing fungi have not been demonstrated. In that case, the luminescence inhibition of *V. qinghaiensis* sp. Q67 by common mycotoxins was investigated. It was found that the luminescence inhibition increased with increasing concentration of mycotoxins after exposure for 15 min, and positive correlations were observed between the mycotoxin concentration and luminescence inhibition with *R*^2^ > 0.98 (Table 1). Li et al. (2012) [21] reported a similar dose-response relationship when fusaric acid was assessed. These results suggested that *V. qinghaiensis* sp. Q67 might be suitable for rapid toxicity assays for various mycotoxins. In this present study, the IC_50_ values of DON, FB_1_, patulin, citrinin, OTA, and ZEN were determined to be 30.74 ± 0.33, 14.88 ± 0.14, 12.23 ± 0.10, 11.02 ± 0.10 and 9.71 ± 0.17 µg mL^−1^, respectively. Among them, DON exhibited the smallest inhibitive activity, while ZEN had the strongest inhibition on the luminous intensity of *V. qinghaiensis* sp. Q67. The first developed bioluminescence assay using *Photobacterium phosphoreum* indicated that the EC_50_ values for toxic effects of ZEN, OTA, and citrinin were 13.51, 16.88, and 20.05 µg mL^−1^, respectively [28]. Bouslimi et al. (2008) reported the cytotoxic effects of OTA and citrinin on kidney cells, with IC_50_ values of 50 µM (20.15 µg mL^−1^) and 220 µM (55.06 µg mL^−1^), respectively [29]. These results suggested that *V. qinghaiensis* sp. Q67 was more sensitive in detecting most mycotoxins than *Photobacterium phosphoreum.* It is well known that different luminescent bacteria have different sensitivities to various mycotoxins. For example, *Aliivibrio fischeri* was used in an acute standard test for aflatoxin B1 with the EC_50_ of 23.3 µg mL^−1^, while *V. fischeri* was applied to the analysis of the same mycotoxin with the EC_50_ of 14.4 µg mL^−1^ [30]. Previous works had proven that mycotoxins such as DON and ZEN have effects on nicotinamide adenine dinucleotide phosphate (NADH) in the bacterial bioluminescence system, thus inhibiting the reaction of FMN oxidoreductase and luciferase [31]. Therefore, it was hypothesized that different mycotoxins could inhibit the *V. qinghaiensis* sp. Q67 with different IC_50_ values were attributed to their different degrees of inhibitory effects on the oxidoreductase and luciferase in the bacterial bioluminescence system, although the reaction mechanism of mycotoxins on the related enzymes has yet to be elucidated.

*V. qinghaiensis* sp. Q67 was used to assess the overall toxicities of the metabolites of 10 fungal pathogens isolated from different tropical fruits. In this study, because sterile CB medium was used as a blank, the effect of the medium can be eliminated. As shown in Table 2, there were good positive correlations (*R*^2^ > 0.95) between the concentration of the culture of *F. proliferatum*, *F. semitectum*, *F. oxysporum*, *F. verticillioides*, *Colletotrichum gloeosporioides*, *Peronophythora litchii*, *Penicillium digitatum* or *Phytophthora infestans*, and the luminescene intensity of *V. qinghaiensis* sp. Q67. However, the toxicities of the metabolites produced by the *Penicillium italicum* and *Geotrichum candidum* were too low to be detected. In addition, we found that *P. italicum* did not produce any toxic compounds (unpublished). Gastélum-Martínez et al. (2013) reported that *G. candidum* did not produce mycotoxins, instead, it inhibited the production of the T-2 toxin when co-cultured with *F. langsethiae* [32]. The genus *Fusarium* is known for production of mycotoxins such as FBs, DON and ZEN [12]. In this study, the IC_50_ of *F. proliferatum* was determined to be 17.49% ± 2.15%, while those of *F. verticillioides* and *F. oxysporum* were determined to be 28.61% ± 5.40% and 33.33% ± 5.67%, respectively. However, *F. semitectum* slightly suppressed the luminous intensity with an IC_50_ of 92.56% ± 11.20%. Thus, *F. proliferatum*, *F. verticillioides* and *F. oxysporum* exhibited higher acute toxicity to *V. qinghaiensis* sp. Q67 than *F. semitectum*. These results were consistent with the mycotoxin-producing ability of these fungi. *F. proliferatum* as a predominant species in maize produced large amounts of FB_1_, beauvericin, and moniliformin, which could explain its high toxicity to *Artemia salina* larvae [33]. According to a report by Mikušová et al. (2013), *F. proliferatum* also produced large amounts of FB_1_ and FB_2_ in postharvest grape berries [34]. However, *F. semitectum* was reported to cause a lower mortality of brine shrimp than *F. proliferatum*, suggesting that the former had a lower level of capability for producing toxic metabolites than the latter [35]. In addition, *Peronophythora litchii* isolated from decayed lychee fruit exhibited a toxicity with an IC_50_ of 69.45% ± 9.24%. Xie et al. (2010) reported four secondary metabolites from *P. litchii*, but these compounds were not toxic to brine shrimp [36]. *Colletotrichum gloeosporioides* and *Phytophthora infestans* caused relatively low inhibitions of the luminous intensity with the IC_50_ values of 115.90% ± 19.13% and 119.51% ± 12.22%, respectively. A similar phenomenon was also reported for *C. gloeosporioides* and *P. infestans* [37,38]. Although these species are terrible plant pathogens, they have not been reported to produce mycotoxins and, thus, only weak suppression of the luminous intensity was observed. 

Some pathogens are well known to produce toxic compounds, such as mycotoxins, which can exert toxic effects on other species including prokaryotes and eukaryotes. These toxicities can be evaluated by *V. qinghaiensis* sp. Q67 so as to estimate the inhibitory factors of fungus. Usually, pathogens produced more than one single inhibitory factor, and two or more inhibitory factors, especially mycotoxins, may have a synergistic effect on other species. Here, in every aqueous phase sample, *V. qinghaiensis* sp. Q67 suffered multiply inhibitory effects from the substrate. This study indicated the potential toxicity of fungal culture using *V. qinghaiensis* sp. Q67, but these factors that affects the luminous intensity of *V. qinghaiensis* sp. Q67, including the identification of toxic compounds, require to be investigated further. 

**Figure 2:**
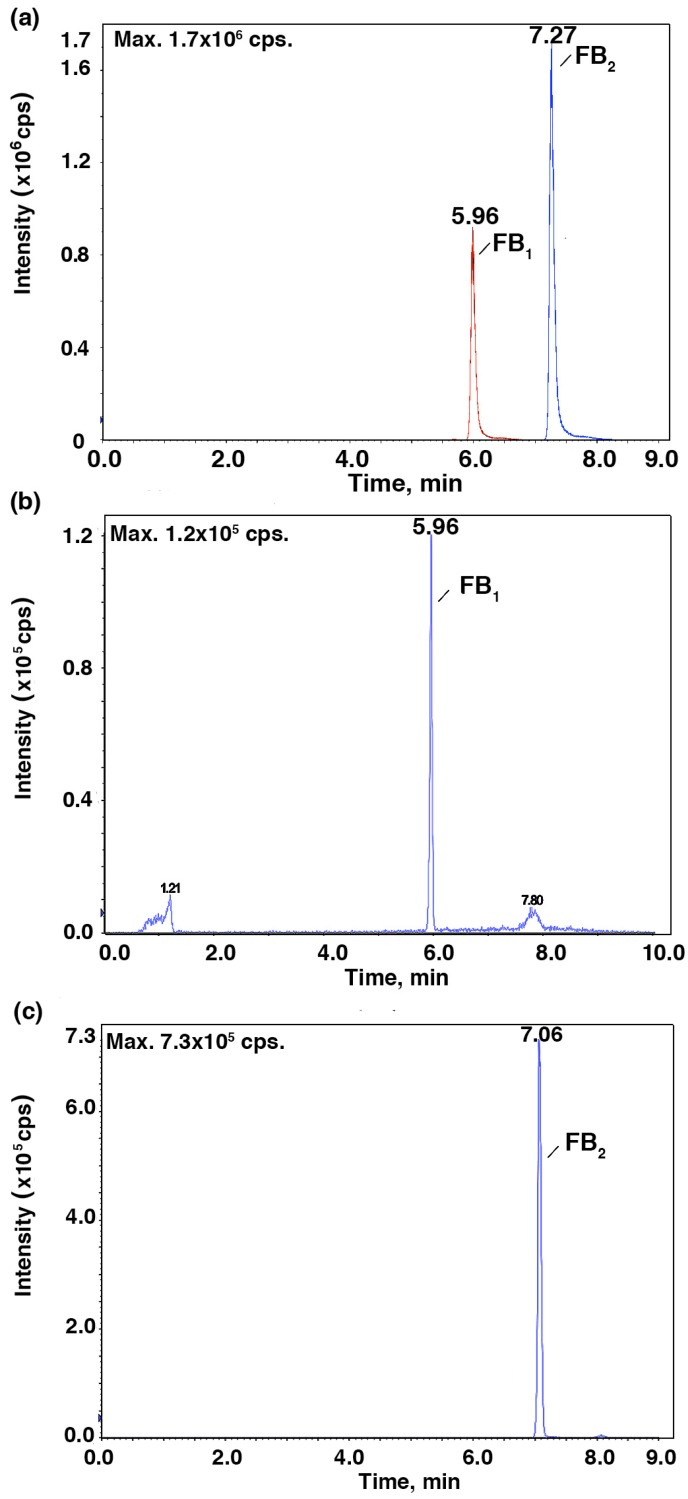
Total ion chromatogram for FB_1_ and FB_2_ at a multiple reaction mode (MRM). (**a**) MS spectra for standard FB_1_ and FB_2_; (**b**,**c**) MS spectra for extract samples. Fumonisins (FBs); mass spectrometry (MS).

**Figure 3:**
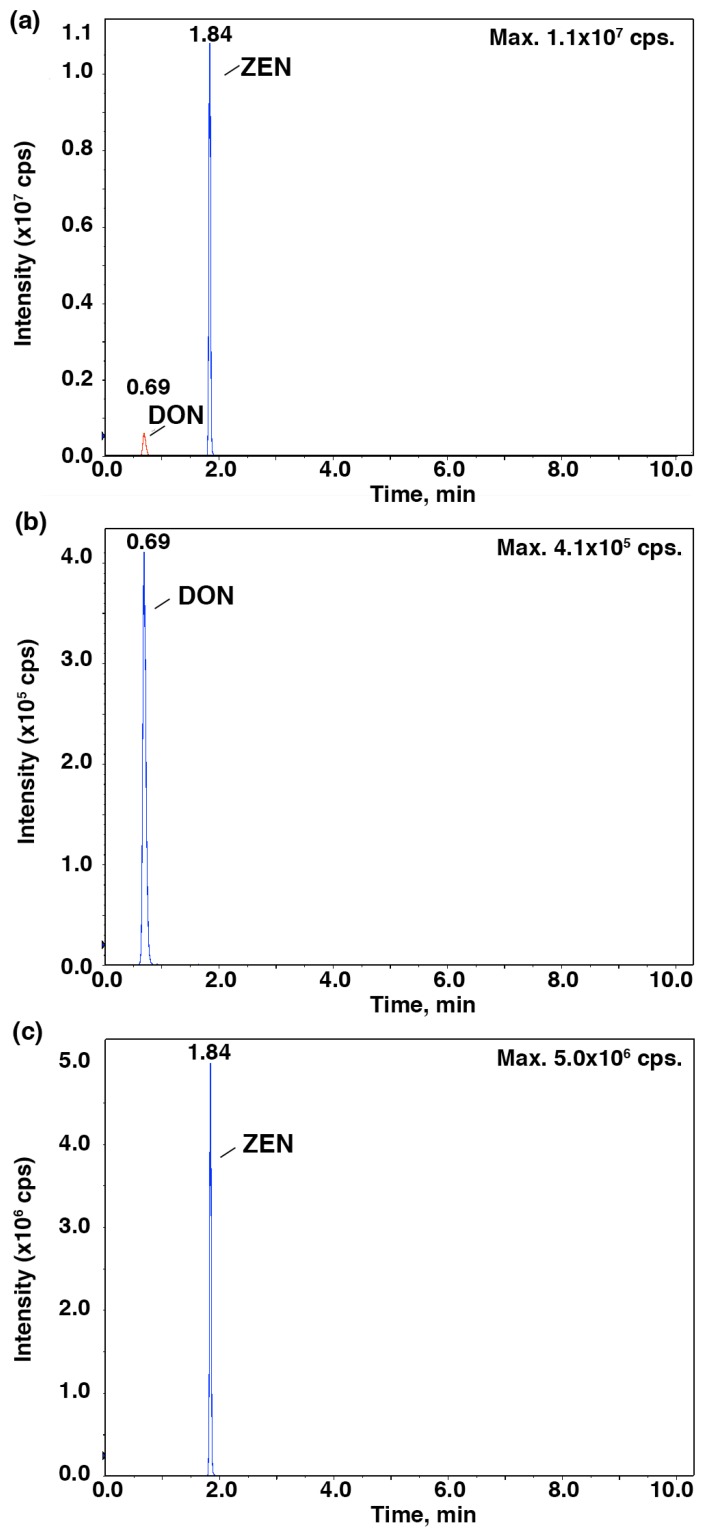
Total ion chromatogram for zearalenone (ZEN) and deoxynivalenol (DON) at a multiple reaction mode (MRM). MS spectra for standard DON and ZEN (**a**); MS spectra for standard DON (**b**); MS spectra for an extract sample (**c**).

**Figure 4:**
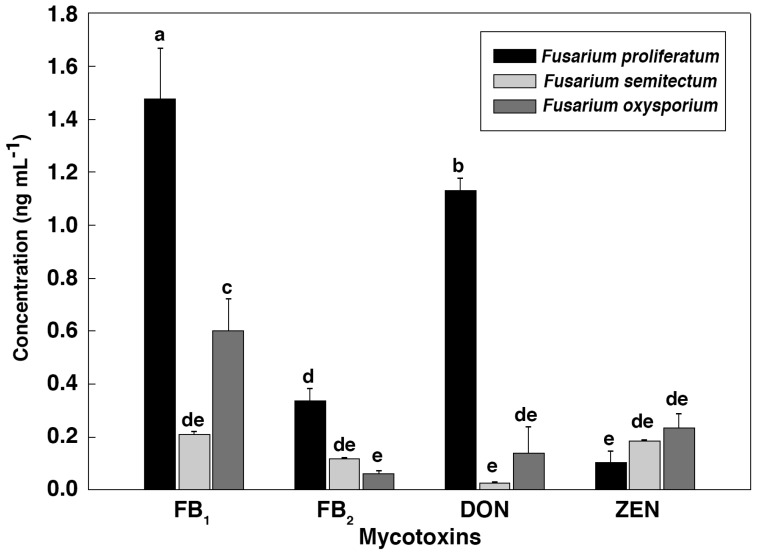
The quantitative analyses of FB_1_, FB_2_, DON and ZEN produced by genus *Fusarium* present in the CB medium by using high-performance liquid chromatography (HPLC)-tandem mass spectrometry (MS/MS). Different letters labeled above columns were significantly different at *p* < 0.05.

To further verify the relationship between the IC_50_ values and the mycotoxin content, HPLC-MS/MS was employed to identify and quantify these mycotoxins from *Fusarium* strains after they were cultured in the CB medium (Figure 2 and Figure 3). As shown in Figure 4, *F. proliferatum* produced the highest amounts of FB_1_ (1.48 ± 0.19 ng mL^−1^), FB_2_ (0.34 ± 0.04 ng mL^−1^) and DON (1.13 ± 0.04 ng mL^−1^). In comparison, *F. oxysporum* had lower amounts of FB_1_ (0.60 ± 0.12 ng mL^−1^) and DON (0.14 ± 0.10 ng mL^−1^) and *F. semitectum* produced the lowest amounts of FB_1_ (0.21 ± 0.01 ng mL^−1^) and DON (0.03 ± 0.00 ng mL^−1^). Yet the amount of FB_2_ produced by *F. semitectum* (0.12 ± 0.01 ng mL^−1^) was higher than that of *F. oxysporum* (0.06 ± 0.01 ng mL^−1^). Furthermore, *F. oxysporum* produced higher amount of ZEN (0.23 ± 0.05 ng mL^−1^) than *F. semitectum* (0.19 ± 0.00 ng mL^−1^) and *F. proliferatum* (0.10 ± 0.04 ng mL^−1^). In general, the amounts of FB_1_ and DON produced by *F. proliferatum* were significantly higher than that of other mycotoxins (*p* < 0.05), which was in agreement with their respective IC_50_ values. In summer, the luminescence tests in the current study showed that the intensity of luminescene negatively correlated with the amounts of mycotoxins, suggesting that *V. qinghaiensis* sp. Q67 can qualitatively assess the toxicity of standard mycotoxins and semi-quantitatively assess the potential toxicity of pathogen’s metabolites especially mycotoxins based on its biological response.

## 3. Materials and Methods 

### 3.1. Chemicals and Solutions

Mycotoxin standards (FB_1_, DON, OTA, ZEN, and citrinin) were purchased from Sigma-Aldrich (St. Louis, MO, USA) while patulin was obtained from R & D Systems (Minneapolis, MN, USA). DON and citrinin were dissolved in methanol and FB_1_, FB_2_, OTA, ZEN and patulin in acetonitrile. Potato dextrose agar (PDA) was purchased from Oxoid Company (Basingstoke, Hampshire, UK). Czapek’s broth (CB) medium was prepared as described by Zhao et al. (2014) [39]. Frozen powder of the luminescent bacterium *V. qinghaiensis* sp. Q67 was provided by Beijing Hamamatsu Photon Technology (Beijing, China). *V. qinghaiensis* sp. Q67 liquid medium was prepared as reported by Jing Li et al. (2012) [21]. All culture media were sterilized at 121 °C for 20 min by autoclaving (Hirayama HVE-50, Tokyo, Japan) before use. 

### 3.2. Fungal Fermentation

Ten strains of fungi isolated from various rotten tropical fruits were kindly provided by Professor Meiying Hu (South China Agricultural University, Guangzhou, Guangdong, China). These fungal strains were firstly grown on PDA plates for 1 week at 28 °C before three agar disks (5 mm in diameter) with full mycelia were cut from each plate and inoculated into 150 mL of CB medium in Erlenmeyer flasks. The flasks were incubated for 4 days on a rotary shaker (200 rpm) at 28 °C in the dark and then the cultures were filtered using the qualitative filter paper (Shuangquan, Hangzhou, Zhejiang, China) to separate mycelia from cultures. The filtered cultures were then centrifuged by using Hettich Universal 32R (Andreas Hettich GmbH & Co.KG, Tuttlingen, Germany) at 15,000× *g* for 3 min at 22 °C to obtain the supernatants, which were diluted with 0.8% NaCl to obtain dilution series for the toxicity bioassay. The original supernatant defined as a concentration of 100% was initially diluted to 50% to test the inhibition of *V. qinghaiensis* sp. Q67 luminescence. If the luminescence inhibition exceeded 50%, the original supernatant was diluted to 5%, 10%, 20%, 40%, 60%, and 80%. Otherwise, the original supernatant was diluted to 20%, 40%, 60%, 80%, and 90%.

*Vibrio qinghaiensis* sp. Q67 was cultured in liquid medium (50 mL) at 22 °C under shaking at 180 rpm in the dark for 16–18 h until it reached the stationary phase. One mL of *V. qinghaiensis* sp. Q67 culture from the flask was transferred to a 1.5 mL centrifuge tube and then centrifuged at 12,000× *g* for 3 min at 22 °C. The supernatant was discarded and then the bacterial pellet was collected and resuspended in 1 mL of 0.8% NaCl solution. The luminous intensity of 0.1 mL of *V. qinghaiensis* sp. Q67 solution was adjusted from 100,000 to 300,000 relative light units.

### 3.3. Luminescence Inhibition Assays

Zinc sulfate heptahydrate (ZnSO_4_·7H_2_O) was dissolved in 0.8% NaCl solution and then used as a reference substrate for testing the luminous intensity of *V. qinghaiensis* sp. Q67. The ZnSO_4_ stock solution was diluted with 0.8% NaCl to 4, 6, 8, 10, 12, 14 and 16 mg L^−1^. Standard mycotoxins were diluted with 0.8% NaCl solution to 5, 10, 15, 20, 25 and 30 µg mL^−1^. 

For toxicity tests, the BPCL-16Y ultra-weak luminescence analyzer, which was provided by Institute of Biophysics, Chinese Academy of Sciences, Beijing, China, was used to measure each sample three times at 1 s intervals at 22 ± 1 °C. For the tested diluent, the luminous intensity of 0.1 mL of *V. qinghaiensis* sp. Q67 solution was initially determined as *I_0_* before it was mixed gently with an aliquot of the reaction liquids (*V. qinghaiensis* sp. Q67 solution:test sample = 1:9, *v*/*v*). After 15 min of reaction, the luminous intensity was determined again and recorded as *I_t_*. All the tests used at the same time interval and each sample was assayed three times. The 0.8% NaCl solution served as a blank control. The luminescence intensities at 0 and 15 min were recorded to be *I_b_*_0_ and *I_bt_*, respectively.

### 3.4. Mycotoxin Extraction

Mycelia were grown on PDA plates as described above. The grown mycelia were inoculated into 150 mL of CB medium in 250 mL flasks and then incubated for 4 days at 28 °C, with shaking at 180 rpm. The culture was filtered through sterile filter paper (Shuangquan Co., Hangzhou, Zhejiang, China). DON and ZEN were extracted using the methods of Li et al. (2005) and Kim et al. (2005), respectively [21,40]. FB_1_ and fumonisin B_2_ (FB_2_) were extracted according to Li et al. (2005) with some modifications. A 10 mL filtrate was mixed with 30 mL of 100% methanol, and then the pH was adjusted between 5.8 and 6.5 using 0.1 M sodium hydroxide solution. A strong anion-exchange column (6 mL, 500 mg) was used to purify the extracts. The supernatant was collected and filtered into an HPLC vial using a 0.22 µm nylon filter (ANPEL Laboratory Technologies Inc., Shanghai, China). The filtrate was stored at −20 °C in the dark prior to the HPLC-MS/MS analysis. To test the rate of recovery, a blank consisting of CB medium was added to standard solutions of FB_1_, FB_2_, DON, and ZEN, and then extracted using the method mentioned above.

### 3.5. HPLC-MS/MS

Mycotoxins were analyzed using an AB-SCIEX TRIPLE QUADTM 5500 HPLC-MS/MS system (AB SCIEX, Framingham, MA, USA). Samples (10 µL each) were injected onto an Ekspert 100 HPLC column (C18 column, 100 × 2.1 mm, 3 µm particle size, ThermoFisher, Waltham, MA, USA) at 35 °C, by the method of Waśkiewicz et al. (2013), with some modifications [41].

For analyses of FB_1_ and FB_2_, the mobile phase of acetonitrile (A) and 5 mM ammonium acetate (B) was used: 10% A for the first 0.5 min, increasing to 50% B for the following 7.5 min, and decreasing to 10% A from 8.0 to 8.5 min. Finally, 10% A was held for an additional 0.5 min prior to return to the initial condition. The pump flow was 0.4 mL min^−1^. For analyses of DON and ZEN, the mobile phase of acetonitrile (A) and 0.1% acetic acid (B) was used. Mobile phase A increased from 10% to 15% for the first 1.0 min, increased to 65% for the next 6.5 min, and then decreased to 10% from 7.5 to 9.5 min. Finally, 10% A was held for an additional 0.5 min before return to the initial condition. The pump flow was 0.5 mL min^−1^. Nitrogen was used as a nebulizing gas at a desolvation temperature of 450 °C. For FB_1_ and FB_2_ analyses, positive electrospray ionization (ESI) [M + H]^+^ was selected for the MS detection to identify the best parameters using a multiple reaction monitoring mode. The parent ion of FB_1_ was set at an *m*/*z* of 722.5, and the daughter ions were set at the *m*/*z* values of 352.4 and 334.4. The parent ion of FB_2_ was set at an *m*/*z* of 706.4, and the daughter ions were set at the *m*/*z* values of 336.4 and 318.4. The mass parameters were optimized for both FB_1_ and FB_2_ analyses using an ion spray voltage of 5500 V, an entrance potential of 10 V, a collision exit potential of 14 V, a cone voltage of 60/65 V, and a collision energy of 50 V. For DON and ZEN analyses, electrospray ionization (ESI) [M − H]^−^ was selected. The parent ion of DON was set at an *m*/*z* of 295.1, and the daughter ions were set at the *m*/*z* values of 265.1 and 138.0. The MS analysis condition for DON included an ion spray voltage of −4500 V, an entrance potential of −10 V, a collision exit potential of −17 V, a cone voltage of −60 V, and a collision energy of −15/−21 V. The parent ion of ZEN was set at an *m*/*z* of 317.2, and the daughter ions were set at the *m*/*z* values of 175.0 and 273.1. The MS analysis condition for ZEN used an ion spray voltage of −4500 V, an entrance potential of −10 V, a collision exit potential of −17 V, a cone voltage of −66 V, and a collision energy of −32/−28 V. The quantification limits of FB_1_, FB_2_, ZEN, and DON were determined to be 0.1 ng mL^−1^. 

### 3.6. Calculation of a Relationship between Concentration and Effect

The luminescence inhibition was calculated according to ISO 11348-3:2007 [42]. When a certain test concentration gives a nearly 0% or 100% inhibition of bioluminescence, the gamma value cannot be calculated because of the edge effect. Therefore, only inhibitory effect value from 10% to 90% was used in the calculation of the relationship between concentration and effect in this study. The inhibition of bioluminescence was calculated using the following formulae. *f* was the correction factor determined by the luminous intensity of the control sample at 0 min (*I*_0_) and after a reaction time of 15 min (*I_t_*):

(1) $$f=I_{bt}/I_{b0}$$

*I_ct_* was the corrected value of *I*_0_:

(2) $$I_{ct}=I_{0}\times f$$

*H_t_* was the inhibitory effect of the test sample and expressed as a percentage:

(3) $$H_{t}=\left[\left(I_{ct}-I_{t}\right)/I_{ct}\times 100\%\right]$$

*Γ* was the gamma value of the test sample after the treatment:

(4) $$\Gamma =[\bar{H}/\left(100\%-\bar{H}\right)]$$

where $\bar{H}$ was the mean of *H_t_*. Depending on *Γ* and *c* (the portion of the test sample as a percentage or the solvent concentration with corresponding measurement unit multiplied by 0.9). Thus,

(5) $$\log_{10}\;c=b\;\log_{10}\;\Gamma +\log_{10}\;a$$

was calculated, where *b* was the value of the slope of the described line while $\log_{10}\;a$ was the value of the intercept of the described line. For the linear regression analysis, the calculated inhibitory effects can be used directly to estimate the parameters of the linear relationship between concentration and effect, from which the IC_50_ value for any level can be derived subsequently. Using the standard least-square regression statistics, the IC_50_ value with corresponding confidence limit can be determined to be *c* = IC_50_ at *Γ* = 1.00. Finally, the relationships between $\log_{10}\;c$ and $\log_{10}\;\Gamma$ were estimated using correlation coefficients (*R*^2^).

### 3.7. Statistical Analysis

All experiments were performed in triplicate. The standard values of all IC_50_ were based on three IC_50_ values calculated from triplicate functions of liner correlation. One-way analysis of variance using SPSS 16.0 software (SPSS Inc., Chicago, IL, USA) was used to determine statistically significant differences. Because multiple comparisons of mycotoxin quantities were used in this study, the calculation of the least significant differences was conducted at *p* < 0.05. 

## 4. Conclusions

Thin-layer chromatography, HPLC, GC-MS, and ELISA are commonly used to detect the major known mycotoxins, such as FBs, aflatoxins, and tricothecenes in diverse samples [2], but their limitations are obvious. For example, the samples must be treated by a series of tedious procedures to extract mycotoxins before tests. Especially for a food matrix, these procedures need to be adjusted according to the different characteristics of the matrix [9,31]. Although these methods are sensitive and accurate, they require expensive equipment and trained personnel, which have limited their applications [6]. In this study, the luminescent bacterium *V. qinghaiensis* sp. Q67 was used to carry out a rapid bioassay to evaluate the potential toxicity of the cultures of mycotoxin-producing fungi. As shown in Table 1, a good correlation (*R*^2^ > 0.98) was obtained between the concentration of fumonisin B1, deoxynivalenol, zearalenone, ochratoxin A, patulin, or citrinin and luminous intensity of *V. qinghaiensis* sp. Q67. In addition, significant correlations between the mycotoxin amount and the luminous intensity from the cultures of 10 major mycotoxin-producing pathogens were obtained. The HPLC-MS/MS analysis confirmed the correlation between the concentration of a mycotoxin and luminous intensity. Thus, *V. qinghaiensis* sp. Q67 as a convenient and rapid test can be used to assess the potential toxicity of fungal secondary compounds prior to their identification to save screening time for these toxic compounds or fungal strains. The method shows great promise to ensure the safety of fungus-associated fresh products, especially tropical fruits. Moreover, this study has laid a foundation to understand the potential toxicity of pathogens in tropical fruits.

## Figures and Tables

**Table 1:** The analyses of the luminescence inhibition of standard mycotoxins.

Mycotoxins	Formula	*R*^2^	IC_50_ (μg mL^−1^)
Fumonisin B_1_	$\log_{10}\;c=0.8854\log_{10}\;\Gamma +1.1727$	0.98	14.88 ± 0.14
Deoxynivalenol	$\log_{10}\;c=0.6969\log_{10}\;\Gamma +1.4877$	0.99	30.74 ± 0.33
Zearalenone	$\log_{10}\;c=0.5599\log_{10}\;\Gamma +0.9871$	0.98	9.71 ± 0.17
Ochratoxin A	$\log_{10}\;c=0.1991\log_{10}\;\Gamma +1.0422$	0.99	11.02 ± 0.10
Patulin	$\log_{10}\;c=0.3160\log_{10}\;\Gamma +1.1241$	0.99	13.31 ± 0.14
Citrinin	$\log_{10}\;c=0.0902\log_{10}\;\Gamma +1.0873$	0.99	12.23 ± 0.10

**Table 2:** The analyses of the luminescence inhibition of the cultured fungi by *V. qinghaiensis* sp. Q67.

Fungus	Original	Formula	*R*^2^	IC_50_/%
*Fusarium proliferatum*	*Averrhoa carambola* L.	$\log_{10}\;c=0.7729\log_{10}\;\Gamma -0.7571$	0.98	17.49 ± 2.15
*Fusarium semitectum*	*Musa nana* Lour.	$\log_{10}\;c=0.3064\log_{10}\;\Gamma -0.0336$	0.98	92.56 ± 11.20
*Fusarium oxysporium*	*Musa nana* Lour.	$\log_{10}\;c=0.3140\log_{10}\;\Gamma -0.4771$	0.98	33.33 ± 5.67
*Fusarium verticillioides*	*Litchi chinensis* Sonn.	$\log_{10}\;c=0.6071\log_{10}\;\Gamma -0.5434$	0.99	28.61 ± 5.40
*Colletotrichum gloeosporioides*	*Mangifera indica* L.	$\log_{10}\;c=0.8040\log_{10}\;\Gamma +0.0641$	0.97	115.90 ± 19.13
*Peronophythora litchii*	*Litchi chinensis* Sonn.	$\log_{10}\;c=0.3446\log_{10}\;\Gamma -0.1583$	0.99	69.45 ± 9.24
*Penicillium italicum*	*Citrus reticulata* Blanco	N/A		
*Penicillium digitatum*	*Citrus reticulata* Blanco	$\log_{10}\;c=0.5050\log_{10}\;\Gamma +0.3294$	0.97	213.50 ± 46.08
*Geotrichum candidum*	*Citrus reticulata* Blanco	N/A		
*Phytophthora infestans*	*Musa nana* Lour.	$\log_{10}\;c=0.3655\log_{10}\;\Gamma +0.0774$	0.99	119.51 ± 12.22
